# RETRACTION: Effects of High Tibial Osteotomy Compared With Unicondylar Knee Arthroplasty on the Surgical Site Wound Infection and Pain in Patients With Medial Knee Osteoarthritis

**DOI:** 10.1111/iwj.70274

**Published:** 2025-03-03

## Abstract

**RETRACTION:** LiJ. , ZhangY. , ChenY. , LiY. , DaiB. , and LiuP. , “Effects of High Tibial Osteotomy Compared With Unicondylar Knee Arthroplasty on the Surgical Site Wound Infection and Pain in Patients With Medial Knee Osteoarthritis,” International Wound Journal 21, no. 3 (2024): e14773, 10.1111/iwj.14773.38477639 PMC10936230

The above article, published online on 13 March 2024, in Wiley Online Library (http://onlinelibrary.wiley.com/), has been retracted by agreement between the journal Editor in Chief, Professor Keith Harding; and John Wiley & Sons Ltd. Following an investigation by the publisher, all parties have concluded that this article was accepted solely on the basis of a compromised peer review process. The editors have therefore decided to retract the article. The authors did not respond to our notice regarding the retraction.

**RETRACTION:** J. Li , Y. Zhang , Y. Chen , Y. Li , B. Dai , and P. Liu , “Effects of High Tibial Osteotomy Compared With Unicondylar Knee Arthroplasty on the Surgical Site Wound Infection and Pain in Patients With Medial Knee Osteoarthritis,” International Wound Journal 21, no. 3 (2024): e14773, 10.1111/iwj.14773.38477639 PMC10936230

The above article, published online on 13 March 2024, in Wiley Online Library (http://onlinelibrary.wiley.com/), has been retracted by agreement between the journal Editor in Chief, Professor Keith Harding; and John Wiley & Sons Ltd. Following an investigation by the publisher, all parties have concluded that this article was accepted solely on the basis of a compromised peer review process. The editors have therefore decided to retract the article. The authors did not respond to our notice regarding the retraction.

